# Which need characteristics influence healthcare service utilization in home care arrangements in Germany?

**DOI:** 10.1186/1472-6963-14-233

**Published:** 2014-05-22

**Authors:** Lena Dorin, Suzi C Turner, Lea Beckmann, Jörg große Schlarmann, Andreas Faatz, Sabine Metzing, Andreas Büscher

**Affiliations:** 1 Graduate School Family Health in the Lifecourse, Faculty of Business Administration and Social Sciences, University of Applied Sciences Osnabrück, Caprivistraße 30a, 49076 Osnabrück, Germany; 2 Department of Nursing Science, Witten/Herdecke University, Stockumer Str. 10, 58453 Witten, Germany; 3 Master of Public Health in Epidemiology Student, Georgia Southern University, 1332 Southern Dr, Statesboro, GA 30460, USA; 4 Quantitative Methods, Faculty of Business Administration and Social Sciences, University of Applied Sciences Osnabrück, Caprivistraße 30a, 49076 Osnabrück, Germany; 5 Nursing Science, Faculty of Business Administration and Social Sciences, German Network for Quality Development in Nursing (DNQP), University of Applied Sciences Osnabrück, Caprivistraße 30a, 49076 Osnabrück, Germany

**Keywords:** Service utilization, Long-term care, Home care arrangements, Needs, Elderly, Family caregivers, Family caregiving, Long-term care services

## Abstract

**Background:**

We see a growing number of older adults receiving long-term care in industrialized countries. The Healthcare Utilization Model by Andersen suggests that individual need characteristics influence utilization. The purpose of this study is to analyze correlations between need characteristics and service utilization in home care arrangements.

**Methods:**

1,152 respondents answered the questionnaire regarding their integration of services in their current and future care arrangements. Care recipients with high long-term care needs answered the questionnaire on their own, the family caregiver assisted the care recipient in answering the questions, or the family caregiver responded to the questionnaire on behalf of the care recipient. They were asked to rank specific needs according to their situation. We used descriptive statistics and regression analysis.

**Results:**

Respondents are widely informed about services. Nursing services and counseling are the most used services. Short-term care and guidance and training have a high potential for future use. Day care, self-help groups, and mobile services were the most frequently rejected services in our survey. Women use more services than men and with rising age utilization increases. Long waiting times and bad health of the primary caregiver increases the chance of integrating services into the home care arrangements.

**Conclusion:**

The primary family caregiver has a high impact on service utilization. This indicates that the whole family should be approached when offering services. Professionals should react upon the specific needs of care dependents and their families.

## Background

Ageing is one of the most demanding challenges of this century especially for industrialized countries [1]. According to the European Union, 20% of the European population will be 65 and over by 2025 [2]. Simultaneously, the number of persons being 80 years and older is increasing faster than any other segment of the population in all EU Member States. Considering that seniors have different and mostly higher healthcare dependencies, adaption of health and long-term care systems is necessary to provide adequate care [3]. The adaption of health and long-term care is necessary considering that the increasing need for formal care, especially institutional care, is restricted due to limited personnel and diminishing care capacities for the heightened care demands. The rising number of older adults increases the likelihood that more formal long-term care services will be utilized, with home care nursing service proving to be the most likely used type of professional assistance [4]. For the users, the national long-term care system is relevant because it defines a general surrounding for the care-dependent and has to be recognized when transferring the results to other countries.

### The German long-term care system

The current long-term care insurance system in Germany is a part of the overall national obligatory social insurance scheme that includes sickness, pension, accidental, unemployment, and lastly long-term care insurance [5]. A person is eligible to receive long-term care benefits if he or she is unable to perform regular activities of daily living in the areas of personal hygiene, nutrition, or mobility due to physical or mental impairment [6]. Severity of one’s situation is divided into three levels. As the individual’s condition worsens and the time to provide tasks by the caregiver increases, the level of care rises and the benefits the care recipients get act correspondingly [7].

Eligibility criteria for the first level of care is described as depending on formal or informal assistance for at least one hour and thirty minutes per day performing the activities of daily living aforementioned [8]. Level two is defined as needing the same type of assistance for at least three hours per day; level three means a need of assistance for at least five hours daily [8]. As the need for assistance rises due to the complexity of care in conjunction with the time required for daily care, the capability of family members to provide this assistance becomes increasingly difficult [9]. Consequently, the utilization of healthcare services is of great concern. Individuals with care level I status use less professional nursing services than those in care level II and III [10]. This confirms the notion that as the severity of the overall condition increases, so does the likelihood to seek help. This pursuit for help occurs not only inside the family or the social network, but includes professional services as well [11].

### Long-term care preferences

Most people in need of assistance prefer to stay at home even when they need assistance in daily living [12,13]. This is confirmed for people with high-level care needs living in Germany [14]. Currently, 2.5 million people in Germany are care-dependent; that is about 3% of the entire German population. Almost 820,000 care-dependent people are at care level II, 63% are cared for at home, and 37% live in nursing homes [10]. Not only do care dependents favor home care arrangements themselves, but it also matches governmental preferences as this type of long-term care is more affordable in comparison to nursing homes [7]. Home care arrangements can vary widely from only informal (mostly family) support to a variety of professional health and long-term care services [1].

This study investigates service utilization in long-term care arrangements at home. It uses a theoretical framework explaining health service use to better understand which factors are associated with the integration of professional services in home care arrangements. Furthermore, it is an evaluation of whether the theoretical assumptions validate the empirical approach in the context of long-term care. Gaining knowledge about relevant influences implies the chance to adapt current services accordingly and strengthen the offers to support people, even those with multi-morbidity, to stay at home for as long as possible.

### The Andersen Model of health service use

The Behavioral Model of Health Services Use, developed by Ronald Andersen, is one of the most internationally referred models in explaining healthcare utilization. In previous studies, the Andersen Model was mostly applied in acute care or in the context of a specific illness [15]. In regards to long-term care, it has not been used widely; especially not with the inclusion of different need characteristics and how they might influence utilization in home care arrangements.

Andersen determines healthcare utilization through three major components – predisposing, enabling, and need factors [16]. Predisposing factors are represented by demographic characteristics such as age, gender, sex, ethnicity, marital status, occupation, and education [15]. Enabling factors involve the financial and organizational influences on the individual’s use of services [17]. The need element of the Andersen model addresses perceived need and evaluated need. Perceived need is defined as how one views his or her own general health, whereas evaluated need is a professional assessment of an individual’s health [15]. Andersen takes a further look into the matter and advances both definitions by integrating a social feature into the description – perceived need should also be determined by social structure and health beliefs [18]. Similarly, a social attribute is included into the definition of evaluated need [16].

The Andersen model suggests that the strongest predictors and central determinants of healthcare service use are perceived and evaluated need [16]. While perceived need assists in discerning healthcare use and health behaviors of individuals, evaluated need is more closely related to the type and amount of treatment that will be delivered to the patient [16]. Bradley and colleagues (2002) expanded the Andersen model by adding a degree and duration characteristic to the need factor, and concluded that these two characteristics assist in determining what type of long-term care one seeks suggesting a direct correlation of need on utilization [19]. The care levels (I, II and III) presented earlier fit this expanded model most appropriately. The care level determines the degree component; the duration is presented by the daily hours of care needs. The care level on its own does not provide sufficient information about specific personal needs, which can vary widely even within a group of patients with the same level of care.

For our focus we will concentrate on the influence of need characteristics on utilization and take into account that the care level as well as the surrounding health and long term care system and home care as type of long-term care is comparable among the study population.

### Need characteristics

People receiving long-term care benefits are usually suffering from chronic illnesses causing instability in their home care arrangements. This increases their risk of emergency situations and hospitalization, and the likelihood that expanded care will be needed after discharge from the hospital [17,20,21]. Ageing increases the risk of suffering from one or more chronic conditions, and often causes dependency and a necessity for assistance in daily life [22]. This multi-morbidity places older adults in an extremely vulnerable position [20].

While need is mostly associated with physical ability, it can also arise from cognitive components [17]. As the assistance required by the care-dependent individual increases, family members and informal caregivers may become incapable of performing caregiving tasks on their own due to physical strain, emotional distress, financial hardship, or their own health status [23]. Especially for families caring for persons with mental disorders, there is also an increasing risk of social isolation [24].

In spite of this evidence, many families and informal caregivers fail or delay to pursue supportive services for a variety of reasons – lack of awareness, reluctance, affordability, or unavailability [25]. Caregiver strains are determinants of service use, e.g. emotional stress is not only an integral feature of service use by informal caregivers, but it also serves as a catalyst for healthcare service utilization among care recipients [26]. Thus, a higher level of psychological suffering in addition to the presence of co-morbid conditions increases the likelihood that one will seek help through professional outside resources [17].

In this paper we focus on people with high-level care needs in long-term home care arrangements, and analyze which factors of emotional strains as well as other need characteristics increase utilization.

## Methods

The Andersen Behavioral Model of Health Services Use is the framework of our study explaining how certain determinants, specifically needs, influence healthcare utilization without referring to a specific context.

In our survey that was initiated in spring 2012 we focused on home care arrangements of people with severe care needs who fell in the care-level II category, which by definition is more than 180 minutes of daily support and assistance were included in the study. It was developed in cooperation with the Bertelsmann Foundation and a major German health insurance company, BARMER GEK, which insures 8.6 million people – about 10% of the German population.

We sent out 2,524 questionnaires and 1,152 care recipients with severe care needs, along with their families answered, thus our research achieved a response rate of 46%. Due to the vulnerable study population, we expected that not all care-dependents were able to answer on their own. As a result, family members could assist or answer substitutionally.

The study participants were informed in the introduction of the questionnaire that participation was completely voluntarily, and all responses to the survey would remain anonymous. Therefore, the participants were instructed to omit any personal information (e.g. name, address, phone- or insurance numbers). With the returning of the questionnaire each individual constituted his or her consent to participate.

This study could not be built upon an existing instrument that included relevant items for this specific context of long-term care. Knowing that almost all care dependent individuals suffer from one or more chronic diseases, we used research conducted by Corbin and Strauss to consider the characteristics of chronic illness [27]. Furthermore we looked at the official German statistic of care dependency to take recent developments into account [10]. We could furthermore profit from the research on different caring arrangements by Büscher [28].

All categories in the questionnaire related to needs including emotional distress, social isolation, emergency situations, overnight hospital stays, and pressure sores were included in this analyses. The frequency of occurrence regarding these categories was assessed in order to determine their influence upon healthcare service utilization in the specific context of homecare arrangements. Our idea of need is parallel with the Andersen model as it explicitly suggests the considerable impact of need on utilization of healthcare services.

Methods of analysis used were descriptive and multivariate. For the descriptive analyses we present how many people in need of care and/or their families are informed about services, and use or reject services. Care recipients’ and/or families’ potential use of services in the future is presented as well.

In the multivariate analysis we used a logistic regression model to test the association between the socio-demographic variables age and gender of the care recipient, and service utilization among long-term care patients living at home and/or their caring family member. This regression model was also conducted to understand which need characteristics influence service utilization among the same population.

The utilization of one or more services was defined as the independent variable while the dependent variable is described as the need characteristic.

We evaluated psychological stress and further need characteristics through the following questions and categories:

Table 1: Independent and dependent variables.

**Table 1:** Independent and dependent variables

**Independent Variables = Need Characteristics**			
**Categories**	**Questions**	**Possible answers**	**Dichotomization**
	**How often did it recently happen that…**		
	**How often did it recently happen that…**		
**Emotional distress**	…you felt that your relatives were overextended or stressed out?	never	
	…you were concerned that your family caregiver will not be able to provide the same care as nowadays?	very rarely	
	…you waited a long time for help or support?		
**Social isolation**	…you felt lonely or left alone?		
	…you missed contact to other people or personal conversations?	sometimes	
**Emergency situations**	…you were frightened that there could be a medical emergency situation?	often	
	…you feared that you are unable to call for help in case of emergency?		
**Hospital stay**	Have you, as a care recipient, been in the hospital overnight in the last 12 months?	no	
		yes, once	
		yes, twice	
		yes, more than twice	
**Length of stay**	How long have you been at the hospital for the past 12 months?	less than one week	
		up to two weeks	
		two to four weeks	
		more than four weeks	
**Pressure sore**	Have you ever had a pressure sore?	yes	
		no	
**Caregiver**	How do you assess the health of your primary caregiver?	bad	
		less well	
		good	
		very good	
		excellent	
**Dependent Variables = Services**			
**Services**	**Explanations**		
**Nursing service**	A nursing service offers professional support in personal care, nutrition, and domestic care	I already use it	use
		→ utilization	
**Counseling**	Counseling can be offered by nursing services, hospital/healthcare insurance, or from community care access centers. It can also take place in the home environment.	I don’t use, but I want to use it	
			no use
**Training and Guidance**	Training and guidance for care recipients and their family caregivers are provided by nursing services, health/care funds – also possible at home	→ potential future use	
**Day care**	At the day center, the day care recipients are cared for in a facility. A pick-up and delivery service is organized as well as meals/food.	I don’t use it and I don’t want to use it	
**Short-term care**	Short-term care is the temporary (maximum of four weeks per year) move to a short-term care facility, for example after a hospital stay, or in case the family care person is not available for a particular time period.		
		→ rejection	
		I don’t know what this is	
**Mobile Services**	Mobile services bring drinks, food, or other purchases directly to your home, e.g. meals on wheels.		
		→ not informed	
**Self-help groups**	This is where relatives of care recipients meet for (regular) exchange.		

### Ethic statement

The Ethical Committee of the German Society for Nursing Science evaluated the proposal for this study. The committee declared that no further ethical approval needs to be obtained. The committee pointed out that the BARMER GEK, as a German statutory health insurance, is a non-profit organization that has the status of a non-departmental public body and underlies special restrictions by German law for data security and protection. All authors worked with anonymous data only. The research is conducted in compliance with the Helsinki Declaration.

## Results

### Service knowledge, utilization and rejection

This descriptive analyses section presents the prevalence of special need characteristics and service utilization. This prepares for the regression analysis, where we tested for associations between need characteristics and utilization.

The top services in long-term care arrangements are nursing services and counseling (see Table 2). In more than half of the arrangements, nursing services offer professional support for the care recipient and the family. The utilization of counseling is almost as high, followed by short-term care used by nearly one in four. More than every fifth care recipient used mobile services. Slightly less gained experience with guidance and training. Approximately one in twenty care recipients visited day care; a slightly higher number met other families in self-help groups.

**Table 2:** Utilization, Potential future use, Rejection and Not informed per Service in % (N)

**Support Services**	**Utilization**	**Potential future use**	**Rejection**	**Not informed**	**Total**
**Nursing Service**	58.1% (548)	13.7% (129)	25.8% (243)	2.4% (23)	100% (943)
**Counseling**	56.9% (510)	18.3% (164)	16.6% (149)	8.2% (74)	100% (897)
**Short-term care**	24.2% (214)	32.3% (286)	36.8% (326)	6.8% (60)	100% (886)
**Mobile Services**	21.0% (188)	13.0% (116)	61.1% (546)	4.9% (44)	100% (894)
**Guidance and Training**	18.0% (152)	26.2% (222)	37.4% (316)	18.4% (156)	100% (846)
**Day care**	5.4% (46)	17.2% (146)	66.1% (560)	11.2% (95)	100% (847)
**Self-help groups**	6.2% (53)	13.7% (118)	58.8% (506)	21.3% (183)	100% (860)

Table 2: Utilization, Potential future use, Rejection and Not informed per Service in % (N).

Table 2 not only describes the utilization of services but it further reports the potential future use, rejection, and non-informed. Initially, 1,152 questionnaires were received but only 846 to 943 of these surveys could be included in the analysis of services. Depending on the service, respondents ranked due to their personal situation.

Short-term care was found to be the service with the highest potential future use for roughly one out of three families – here we see a higher number suggesting to use this service in the future in comparison to the experienced utilization. We see this same trend again in day care.

Even though more than every second person received advice and information through counseling, there is still almost one in five respondents that prospectively considered using the service. Potential future use was also seen as being higher than the current utilization in both the self-help and guidance and training services. The service with the lowest potential use of only 13% was seen in mobile services.

For day care the potential future use of 17% was three times higher than the number of persons who visited this service already. Two in three respondents answered that they do not use day care and do not want to do so in the future; consequently, rejection is three times higher than utilization and potential future use combined. The same trend can be seen for mobile services.

The percentage of responders rejecting nursing services is smaller in comparison to those who currently use the service, but still accounting for one in four responders. About 14% announced a potential future utilization.

People are widely informed about the different services that are offered. Only about 2% of users responded as not being informed about nursing services. Self-help groups as well as guidance and training were the least known services with one of five responders answering that they did not know these services were offered.

Besides the high rejection rate for some of the services, the potential future use shows the opportunity for families to increase professional support in the future.

### Age and gender related differences in utilization

**Figure 1:**
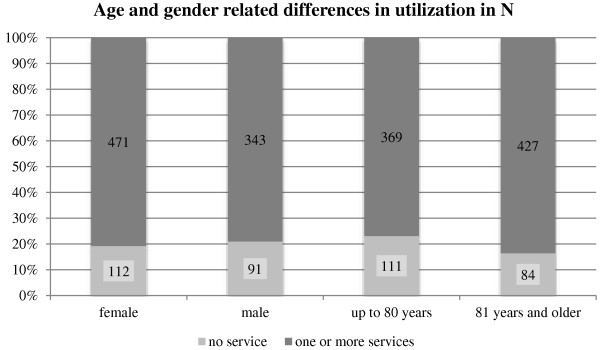
Age and gender related differences in utilization.

The following graph illustrates that only one family in five has no experience in service utilization at all. More than half of the individuals in all groups – female, male, up to 80 years old, and 81 years and older – use one or more services. The care recipients being 81 years and older had a higher rate of using one or more services in comparison to their younger counterparts (Figure 1).

Overall, female care recipients use more services (80.8%) than male (79.0%) but the chi-square test shows that this difference is not significant (chi-square: p = 0.48). The older age group on average utilizes more services (83.6%) than the younger respondents (76.9%). This difference is significant (chi-square: p = 0.003).

### Occurrences of emotional distress, social isolation and emergency situations

Table 3 shows the prevalence of specific needs among care dependents who require more than three hours of support and assistance daily.

**Table 3:** Descriptive analysis of need characteristics

**Categories**	**Questions**	**Answer options**				
	**How often did it recently happen that…**	**never**	**very rarely**	**some-times**	**often**	**total**
**Emotional distress**	…you felt that your relatives were overextended or stressed out?	26.8% (261)	26.5% (258)	38.6% (376)	8.0% (78)	100% (973)
	…you were concerned that your family caregiver will not be able to provide the same care as nowadays?	40.2% (396)	18.9% (186)	32.0% (316)	8.9% (88)	100% (986)
	…you waited a long time for help or support?	72.5% (697)	19.7% (189)	6.2% (60)	1.6% (15)	100% (961)
**Social isolation**	…you felt lonely or left alone?	43.3% (430)	24.6% (244)	25.7% (255)	6.4% (63)	100% (992)
	…you missed contact to other people or personal conversations?	35.0% (346)	23.3% (230)	29.7% (294)	12.0% (119)	100% (989)
**Emergency situations**	…you were frightened that there could be a medical emergency situation?	22.4% (217)	25.4% (246)	41.1% (399)	11.1% (108)	100% (970)
	…you feared that you are unable to call for help in case of emergency?	45.8% (439)	28.5% (273)	20.1% (193)	5.6% (54)	100% (959)

Table 3: Descriptive analysis of need characteristics.

Almost every second respondent (47%) disclosed to sometimes or often feeling that his or her relatives are stressed out or overextended. Only one out of four persons reported never feeling this way. While only about one in twelve respondents frequently waited a long time for help or support, the emotional distress concerning the availability of their family caregiver for future care sometimes or often causes uneasy feelings in over 40%. Emotional distress is data, which has been surveyed from both the caregiver and the care-recipient. From the perspective of the caregiver, he or she may feel that they are losing strength and feeling an increased amount of strain, while the care-recipient may be experiencing feelings of guilt.

The health of the primary caregiver was assessed as bad by less than 5%, but almost every third respondent ranked it as less well (28%). Almost every second respondent answered that the health status of his caring family member is good; approximately one in seven assessed it as very good or excellent.

A majority of respondents, 73%, reported that they never waited a long time for help or support. Forty-one percent found themselves sometimes frightened that there could be a medical emergency situation. Although almost every other person never feared that they would be unable to call for help in the case of an emergency, one in four individuals exhibited this worry sometimes or even often.

Another risk is hospitalization. Only 30% of the patients with high-level care needs have not been in hospital overnight during the last 12 months. Relatively the same number of patients has been in the hospital once during that time period; 23% stayed in the hospital twice. More than one in six persons had more readmissions to deal with. One further need characteristic of institutional care is the duration; almost 29% stayed in the hospital during the last year for more than a month. A similar number of persons, 27%, stayed in the hospital for 2 to 4 weeks. 22% of care dependents had shorter stays of up to two weeks. About the same number only experienced shorter stays of less than a week. A quarter of individuals revealed that occasionally they felt lonely; a similar number of persons missed contact with other people or intimate conversations.

A particular event that shows a very high dependency upon assistance from caregivers is pressure sores. Pressure sores can only be prevented and must be treated by re-positioning the care recipient on a regular schedule. One out of six care recipients has experienced a pressure sore.

### Correlations between need characteristics and service utilization

All factors we presented show specific needs in long-term care arrangements. The Andersen Model suggests the more needs one has, the higher the chance of service utilization. We built a regression model using all presented need characteristics as independent variables and tested for correlation with service utilization as dependent variable.

Table 4: Correlations between need characteristics and service utilization.

**Table 4:** Correlations between need characteristics and service utilization

**Need characteristics as independent variable**	**Use of one or more services as dependent variable**		
	**OR**	**Confidence-Inteval**	**Chi-square: p-value**
**Overextension or stress of family caregivers**	2.19	0.58-11.37	0.256
**Availability of same informal care in the future**	0.29	0.055-0.915	0.034*
**Long waiting-time for help or support**	10.23	1.907-116.353	0.004*
**Feeling lonely or left alone**	0.48	0.087-2.20	0.346
**Missing contact with other people or personal conversations**	3.10	0.77-18.09	0.115
**Frightened of a medical emergency situation**	0.93	0.29-2.75	0.892
**Feared being unable to call for help in case of emergency**	0.79	0.21-2.88	0.715
**Hospital stay in the last 12 months**	0.48	0.11-2.88	0.250
**Duration of hospitalization**	0.81	0.29-2.27	0.687
**Occurance of pressure sore**	0.47	0.03-7.14	0.584
**Health of your primary caregiver**	6.34	1.768-54.298	0.001*
**Gender**	0.13	0.01-1.22	0.078
**Age**	641.30	9.141-694517.9	0.0003*

AIC (Akaike’s information criterion) = 66.32; Deviance = 38.32 (p = 0.0003, Chi-square).

*significant, a p-value of <0.05 was chosen as being significant.

We tested three different groups: care dependents answering on their own, family members alone, and family members answering together with the care dependent. There are only significant correlations in the group of the independently answering care dependents. Three need characteristics and one socio-demographic characteristic show significant correlation with utilization. Age (separated in two groups of up to 80 years and 81 years and older) shows a significant correlation (p = 0.0003). As did the need characteristics items on: caregivers health (p = 0.0014), availability for future caregiving (p = 0.0336) and long waiting time for help and support (p = 0.0043).

The health of the caregiver and his or her ability to provide future care operate as need components of the model. As the results indicate, the health of the family caregiver significantly raises the chance to utilize available healthcare services (OR = 6.34). If the future availability of the primary caregiver is doubted, the chance to include professional home care decreases, as the odds ratio (0.29) shows. The results further show that a person placed at care level II who waits a long time for help or support is more likely to use professional healthcare services (OR = 10.23). The results of this study suggest that there is a correlation between emotional strains and healthcare utilization among long-term care patients.

## Discussion

The majority of people with long-term care needs remain at home; family members mostly care for their relatives. With rising demands and dependency, professional services are included more often in a lot of home care arrangements [10]. Most often this is home care or counseling. Utilizing a nursing service not only relieves the family caregiver but might also alleviate the feelings of guilt perceived by the care recipient [29]. Utilization of professional services may also increase to create a stable care atmosphere and avoid moving to institutionalized care – thus reducing stress upon the care recipient.

Day care and self-help groups gain less attention among the care dependents and his or her family. There are high rejection rates among all services ranging from one in six for counseling to two in three for day care.

The rejection of guidance and training was approximately twice as high as the utilization implying that the families doubt the helpfulness of such services or other barriers exist. These barriers may include access, finances, or lack of a recommendation by a professional/specialist. The same trend was identified for self-help groups. In this case the rejection was nearly twelve times greater than utilization. Perhaps the reason for this is that time and resources to organize supplemental care are limited. Only when individuals anticipate that the service will be of some benefit they will put effort towards overcoming the barriers [26].

We see high rejection rates for day care, even higher among women than men. The reasoning behind this trend could be traced back to the personal history of men. While men are accustomed to being away from the home for their typical work, older women more often worked at home and have shown to use less services offered by institutions which take place away from their known surrounding.

Besides the high rejection rate for some of the services, the potential future use shows the opportunity for families to increase professional support in the future. Potential future care may provide a feeling of security for the families. When the situation gets more complex they have opportunities to include more professional services into the home care arrangements. The issue that arises is that the care dependent and/or their families may wait too long to involve services with the situation bearing the risk that it can only be stabilized in an institutional setting.

Potential use for four out of the seven services was higher than utilization.

The highest potential future use is seen in short-term care. Almost every third respondent answered that he has not used this service so far but that he might do so in the future.

Although all individuals require more than three hours of daily support, their use of services varies from absolutely no utilization to low or heavy utilization; with women significantly using more services than men. One reason might be that most of the husbands have died and children usually have other duties such as work or care for their own children.

### Discussion of the regression analysis

As the Andersen Model suggests, some need characteristics in our study were found to have a significant effect on utilization. Only four out of eleven need characteristics we integrated in our model significantly increase the chance for professional healthcare service utilization.

The strong dependency of people with high-level care needs dominate the regression model. When the care dependent doubts the availability of the family caregiver for future assistance, it decreases the chance of utilizing professional support services in the ambulant setting. A reason for this might be, that if the health of the primary caregiver is getting worse the home care arrangement as a whole is questioned. There might be a higher probability of relying on institutional care because professional support at home cannot replace the support of a family caregiver.

Because of their dependency, the absence of a caregiver, or fear thereof, causes anxiety in people with long-term care needs [30]. There are probably various reasons for utilizing professional services when there is emotional distress. One wish might be to protect the family caregiver from further exhaustion due to support provided by professional services.

Age is a predisposing factor according to the Andersen Model, but in the context of long-term care it can also be rated as a further need characteristic as it normally increases the difficulties in daily living. Difficulties are due to debility of sight or a hardness of hearing which increases dependency in daily living. As one gets older these limitations due to rising age occur and in addition the likelihood of suffering from one or more chronic conditions increases. Resultantly, the probability that the individual will utilize more services increases which is evident in this study.

In contrast to age, gender does not show a significant correlation but it was shown that women use more services than men. The care modalities and health-related factors were more sensible predictors of utilization [31].

Seven out of eleven need characteristics did not show such a significant impact. This does not fully support the Andersen Model but underlines research attempting to work with this model in the context of long-term care. Needs are not constant and one need can be very demanding one day and totally subordinated the next day. The situation of families in long-term care arrangements is not stable but due to the context of multi-morbidity and high dependency it varies a great deal and bears major challenges. We confirmed that age increases utilization [15]; additionally, the strains of long waiting times for help and support and the health condition of the primary caregiver has to be taken into account for rising utilization [9]. When the Andersen Model is adapted to home care arrangements in long-term care it is necessary to not only look at the care dependent but take into account the family perspective with special attention to the primary care giver.

### Further implications

This study reveals that despite the involvement of a professional service in four out of five home care arrangements, many services have high rejection rates.

Further research is needed to understand the barriers leading to the high rejection rate. Strains in home care arrangements will be reduced only if services are developed according to the needs of the care dependents and their families, especially emotional strains. Emotional support can also be provided from professionals [23]. As we know, family members balance the profit they might receive in daily life before they allow professionals to enter their home [28]. For some services we see high potential future rates of service utilization. The potential future use might even be higher when the care recipient or the family member is in a situation where they must begin to use a certain service that they did not utilize before due to progress of the illness or changes in the caring arrangement itself and reacts with informing and, after that, using a special service that he or she might not have known before. For those services the low-level first access has to be organized because intensifying the utilization of a known service is easier than integrating a new one. If professional support can be easily expanded, it can help to stabilize situations and institutional care is less often the only and last opportunity for caring for people with high-level long-term care needs. However, the professional services have to react upon the specific needs of the care dependent and their families. Such need-analyses have to be taken into account when developing, caring, and supporting concepts as well as shaping the design of services. By utilizing home care services, seniors are not only able to improve their functional abilities but also the use of their own resources, resultantly promoting independency [22].

As this is the first study in this specific context, there is only limited data the findings can be compared to. The BARMER GEK is only one, yet the biggest health insurance provider. Data obtained from BARMER GEK is in comparison to other public health insurance data the most representative of the German population [32].

The combination of multi-professional research teams with qualitative and quantitative research experience, epidemiologic knowledge, and a phenomenological perspective is necessary to understand the barriers and needs of long-term care patients and their families [30]. Furthermore, professionals must collect additional information regarding specific needs and promote early inclusion of professional services. Early use of professional services not only assists in avoiding institutional care but releases family caregivers from strains and overwhelming responsibilities, as well as relieving care dependents from feelings of guilt. This is not only an implication for Germany. To deal with the demographic developments of industrialized countries, and the rising number of long-term care patients, an allocation of more resources towards professional care may be necessary. The results are useful for long-term care planning purposes in politics and for the future design of supporting services.

## Conclusions

The context of long-term home care arrangements might be so complex that we have to be careful with the assumption that needs are most relevant for utilization, as the Andersen Model suggests. In the context of health care studies based on the Andersen Model showed that enabling characteristics, population characteristics and external factors influence utilization as well. Perhaps in the context of long-term care negotiation between family caregivers and their care dependent regarding whether the possible benefit is higher than the costs in form of privacy and organizational work has more influence in the context of long-term care arrangements. This is an interesting topic for further research.

## Competing interests

The authors declare that they have no competing interests.

## Authors’ contributions

LD conducted the study (development of questionnaire, data analysis) as part of her PhD. ST supplemented the literature analyses and revised language and spellings. LB revised the manuscript. JGS and AF supported the statistical analyses. The study is supervised by AB, who also contributed to the questionnaire and the manuscript. SM revised the manuscript. All authors read and approved the final manuscript.

## Pre-publication history

The pre-publication history for this paper can be accessed here:

http://www.biomedcentral.com/1472-6963/14/233/prepub
